# Full shell coating or cation exchange enhances luminescence

**DOI:** 10.1038/s41467-021-26490-7

**Published:** 2021-10-26

**Authors:** Yi Zhang, Pengpeng Lei, Xiaohui Zhu, Yong Zhang

**Affiliations:** 1 Department of Biomedical Engineering, Faculty of Engineering, National University of Singapore, Singapore City, 117583 Singapore; 2 School of Environmental and Chemical Engineering, Shanghai University, Shanghai, 200444 China

**Keywords:** Nanoparticles, Nanophotonics and plasmonics

## Abstract

Core-shell structure is routinely used for enhancing luminescence of optical nanoparticles, where the luminescent core is passivated by an inert shell. It has been intuitively accepted that the luminescence would gradually enhance with the coverage of inert shell. Here we report an “off-on” effect at the interface of core-shell upconversion nanoparticles, i.e., regardless of the shell coverage, the luminescence is not much enhanced unless the core is completely encapsulated. This effect indicates that full shell coating on the luminescent core is critical to significantly enhance luminescence, which is usually neglected. Inspired by this observation, a cation exchange approach is used to block the energy transfer between core nanoparticle and surface quenchers. We find that the luminescent core exhibits enhanced luminescence after cation exchange creates an effective shell region. These findings are believed to provide a better understanding of the interfacial energy dynamics and subsequent luminescence changes.

## Abstract (web summary)

Core-shell designs enhance the luminescence of lanthanide-doped upconversion nanoparticles (UCNPs), but the effect of shell coverage was insufficiently characterized. Here the authors demonstrate, on a series of core-shell UCNPs with various shell coverage ratios, an on-off effect by which luminescence is enhanced only when a full coverage is achieved.

## Introduction

Lanthanide-doped nanocrystals have been extensively investigated due to their great promise in multiplexing sensing^1–3^, photovoltaics^4–6^, LED (light emitting diode) display^7–9^, etc. Among them, lanthanide-doped upconversion nanoparticles (UCNPs) represent a class of optical materials that are capable of transforming low energy excitation into high energy luminescence^10,11^. Particularly, benefiting from the ability of upconverting long-wavelength radiation to short-wavelength emission, lanthanide-doped UCNPs have drawn increasing interests for biological applications because of the deeper tissue penetration depth, weaker background interference, and fewer photodamage endowed by the long-wavelength excitations^12–14^.

Typically, lanthanide-doped UCNPs are doped with two types of ions, sensitizers (e.g., Yb^3+^ and Nd^3+^) and activators (e.g., Er^3+^, Tm^3+^, and Eu^3+^), of which sensitizers absorb the incident light and transfer excitation energy to activator for luminescence emission. However, for the small-sized UCNPs, the total surface area per unit volume of the particle would increase as the particle size decreases, which may provide more luminescent quenching centers (e.g., surface defects, surface oscillators, etc.)^15^. In light of this issue, various approaches have been proposed in order to improve the luminescence of nanosized UCNPs, including dye-sensitization strategy^16,17^, coupling with surface plasmon^18,19^, local structural engineering^20–22^, etc.^23,24^. Nevertheless, construction of core-shell architecture is still considered as one of the widely used methods to enhance luminescence of UCNPs^25^. For example, Zhou et al. recently reported a core-multi-shell design to alleviate crores-relaxation and promote interfacial energy transfer process, which achieved an upconversion luminescence quantum yield (UCQY) of 6.34% (4.5 W cm^–2^)^26^. Homann et al. proposed to use anhydrous rare-earth acetates to prepare core-shell UCNPs (45 nm) and reported a UCQY value of 9% (30 W cm^–2^), which was close to that of the bulk counterpart (10.3%) at the same power density^27^.

As for the mechanism of luminescence enhancement via core-shell engineering, Wang et al. firstly reported that coating of a thin inert shell could retain the optical integrity of luminescent core and also effectively reduce emission losses caused by the surface quenching effects^28^. Later, it was proved that the inert shell could decouple the surface and concentration quenching processes for colloidal lanthanide-doped nanocrystals, thus allowing for high-dopant concentrations in the luminescent core^29^. More recently, time-resolved spectroscopic studies indicate that the surface of UCNPs can be gradually passivated with the thickness of inert shell. However, when the UCNP surface is totally passivated, further increasing shell thickness would lead to slight decrease in the emission intensity due to some potential non-radiative quenching pathways from the interface^30^. Based on traditional understanding, it has been intuitively accepted that coating of an inert shell can act as a protective barrier that shields the luminescent UCNP core from its surrounding quenching centers. Nevertheless, the underlying picture of surface quenching dynamics through core-shell interface has not been well elucidated. Despite some discussion of this issue^29^, there are very few studies that directly report a synchronous correlation between a step-wise surface passivation and resulting luminescence change for the core-shell UCNPs. To address these issues, it is necessary to prepare core-shell UCNPs with different shell coverage and to study its relationship with energy transfer and luminescence emission.

In this work, core-shell UCNPs with various shell coverage ratios are demonstrated using silica coated Janus nanoparticles as a template. By increasing the shell coverage, the luminescence intensity of the UCNPs is not much enhanced unless the core nanoparticle is completely covered by the inert shell. In another word, significant enhancement in the luminescence intensity is only observed when a full shell coating is applied. This “off-on” effect in the core-shell UCNPs has not been systematically characterized before and the influence of the shell coverage on the luminescence emission has long been neglected. Furthermore, our time-course study shows that both the emission intensity and color of the highly doped UCNPs vary as the full shell thickness increases. Based on this discovery, a simple post-synthesis approach via cation exchange is developed to eliminate the surface-energy transfer from the core nanoparticle to surface quenching centers, which results in a similar luminescence enhancement effect as the full shell coating. These findings are believed to offer fundamental insights into the mechanisms and process of surface quenching and inspire the design of novel nanostructures with much enhanced luminescence.

## Results

### Synthesis of NaErF_4_/SiO_2_@NaYF_4_ Janus nanoparticles

**Fig. 1:**
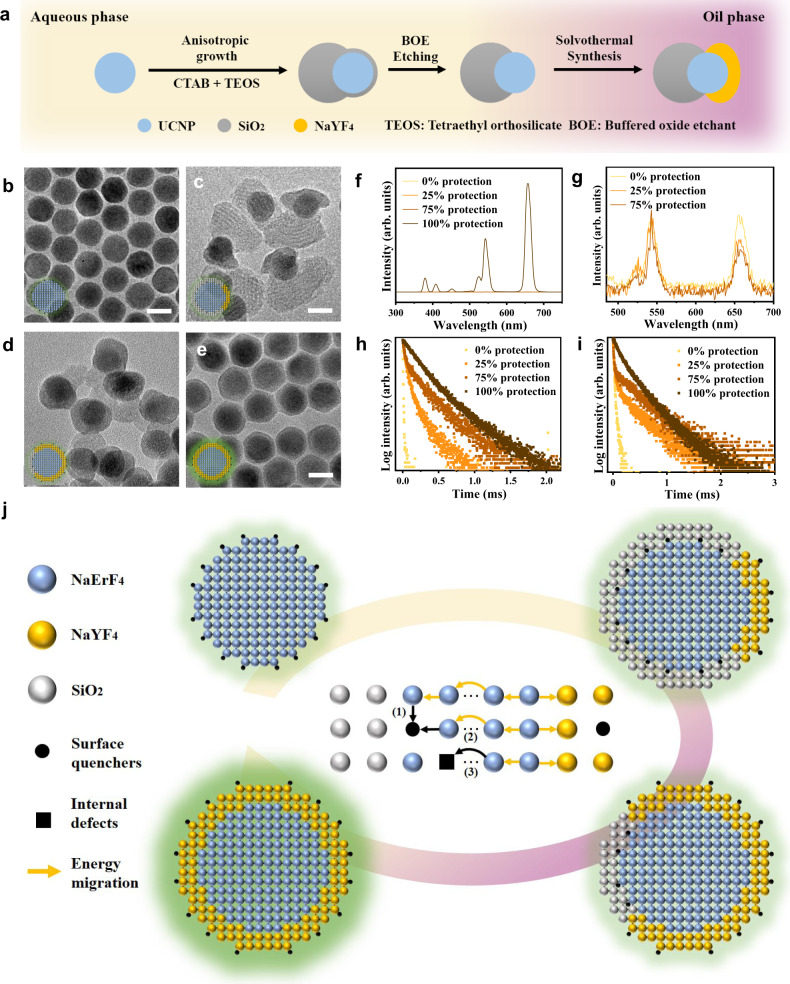
Synthesis and spectroscopic characterization of core-shell structured NaErF_4_/SiO_2_@NaYF_4_ Janus nanoparticles with controllable coverage ratio of NaYF_4_ inert shell. **a** Schematic showing the synthesis process of core-shell structured NaErF_4_/SiO_2_@NaYF_4_ Janus nanoparticles. The surface of NaErF_4_ UCNP (blue) was anisotropically coated by a hydrophilic SiO_2_ shell (gray) via hydrolysis of TEOS (tetraethyl orthosilicate). After etching treatment by BOE (buffered oxide etchant), part of the SiO_2_ shell was removed and the exposed NaErF_4_ surface was used for the growth of hydrophobic NaYF_4_ shell (yellow). **b**–**e** TEM image of NaErF_4_ (0% protection), core-shell structured NaErF_4_/SiO_2_@25%NaYF_4_ (25% protection), NaErF_4_/SiO_2_@75%NaYF_4_ (75% protection), and NaErF_4_@NaYF_4_ (100%protection). Scale bar: 20 nm. **f** The upconversion emission spectra core-shell structured NaErF_4_/SiO_2_@NaYF_4_ Janus nanoparticles with 0% protection, 25% protection, 75% protection and 100% protection upon excited by 980 nm laser light (3 W cm^−2^). **g** Comparison of upconversion emission spectra core-shell structured NaErF_4_/SiO_2_@NaYF_4_ Janus nanoparticles with 0% protection, 25% protection, 75% protection and 100% protection in the spectral range from 500 nm to 700 nm. **h**, **i** Decay curve at 542 nm, and 658 nm of core-shell structured NaErF_4_/SiO_2_@NaYF_4_ Janus nanoparticles with 0% protection, 25% protection, 75% protection and 100% protection. **j** Schematic showing the change at composition, luminescence and energy quenching pathways of core-shell structured NaErF_4_/SiO_2_@NaYF_4_ Janus nanoparticles with an increased NaYF_4_ shell protection.

In order to gain insights into the correlation between surface energy dynamics and luminescent properties, we have specifically developed an approach to synthesize core-shell structured NaErF_4_/SiO_2_@NaYF_4_ Janus nanoparticles with tunable shell coverage (Fig. 1a). In a typical procedure, monodispersed β-NaErF_4_ UCNPs were firstly synthesized using a co-precipitation method (Fig. 1b)^31^. Subsequently, a dense silica layer was coated anisotropically on the NaErF_4_ core by a surfactant-templating method, forming a Janus nanoparticle containing a hydrophobic NaErF_4_ core and a hydrophilic SiO_2_ shell. Afterwards, part of the SiO_2_ shell was removed by buffered oxide etchant (BOE), exposing more hydrophobic surface for further growth of the NaYF_4_ shell.

As illustrated in Fig. 1a, asymmetric coverage of SiO_2_ shell on the NaErF_4_ core is a prerequisite for the fabrication of NaErF_4_/SiO_2_@NaYF_4_ Janus nanoparticles. In this study, a reverse microemulsion method was used, in which cetyltrimethyl ammonium bromide (CTAB) was employed as the micellar template and tetraethyl orthosilicate (TEOS) as the silicon precursor. This approach is employed because highly concentrated CTAB surfactants (3 mg mL^−1^) in the water-oil mixture can lead to the aggregation of CTAB micelles on one side of the UCNP surface, and therefore offer nucleation sites for the growth of SiO_2_ shell through hydrolysis of TEOS precursors. Moreover, the morphology and SiO_2_ coverage can be precisely tuned by varying the amount of TEOS precursors added. For example, both the SiO_2_ coverage ratio (Supplementary Fig. 1a−e) and average hydrodynamic diameters (Supplementary Fig. 1f) can be gradually increased via the addition of TEOS precursors.

Based on the construction of NaErF_4_/SiO_2_ Janus nanoparticles, we verified the feasibility of the direct growth of NaYF_4_ shell on the NaErF_4_/SiO_2_ nanoparticles. Supplementary Fig. 2a and b present the TEM images of NaErF_4_/SiO_2_ Janus nanoparticles with SiO_2_ coverage ratio of 0% and 100%. As shown in Supplementary Fig. 2c, the NaYF_4_ shell can grow uniformly on the bare NaErF_4_ core (i.e., 0% SiO_2_ coverage) and form a core-shell structure. However, for the silica-coated NaErF_4_ nanoparticle (i.e., 100% SiO_2_ coverage), the NaYF_4_ phase does not grow on it but instead nucleates separately (Supplementary Fig. 2d). This is because silica tends to form a thin layer around NaErF_4_ nanoparticle at the beginning of hydrolysis process and cover the whole particle surface over time (Supplementary Fig. 1a−d). Notably, the hydrophilic SiO_2_ coating formed on NaErF_4_ core would prohibit the growth of hydrophobic NaYF_4_ layer. Therefore, the undesired thin portion of the SiO_2_ shell should be removed in order to expose some of the NaErF_4_ core surface for the growth of NaYF_4_ shell.

Although hydrofluoric acid (HF) is a well-known silica removal etchant, it is a highly corrosive liquid and may cause potential risks. Besides, its strong etching ability makes it challenging to precisely control the etching speed. As an alternative, buffered oxide etchant (BOE), a commercial etching mixture of NH_4_F and HF, was used to etch the thin region of SiO_2_ shell from the NaErF_4_/SiO_2_ Janus nanoparticles at a stable etching speed (800 Å min^−1^ at 20 °C). Supplementary Fig. 3 presents the TEM images of NaErF_4_/SiO_2_ Janus nanoparticle with different SiO_2_ coverage ratios (25%, 50% and 75%) after BOE etching, clearly indicating the size and morphology of UCNP nanoparticles are not affected after BOE treatment. More importantly, the hydrophobic side of UCNP nanoparticle has been already exposed via BOE etching process (indicated with red arrow in Supplementary Fig. 3) while the other side is still covered by the SiO_2_ shell. Besides, the coverage ratio of SiO_2_ shell (Supplementary Fig. 4a) and hydrodynamic size (Supplementary Fig. 4b) both gradually decrease with the increase of BOE solution. The averaged zeta potential values for the NaErF_4_@SiO_2_ Janus nanoparticles before and after BOE etching are measured to be −39.97 $\pm$ 2.08 and −16.57 $\pm$ 0.29 mV (Supplementary Fig. 4c), further confirming the partial removal of negatively charged silica. It is worth to mention that no evident change in the upconversion luminescence profiles of UCNPs is observed before and after BOE etching (Supplementary Fig. 4d), indicating the BOE etching has little influence on the upconversion luminescence.

Following the successful etching process, the NaErF_4_/SiO_2_ Janus nanoparticle were obtained and used as seeds for the growth of NaYF_4_ shell. Notably, NaYF_4_ shell is critical in enhancing the luminescence emission, while SiO_2_ shell provides little protection as it can not passivate surface defects of luminescent core^32^. Here, the protection ratio of NaYF_4_ shell is defined as follows:

1 $${{{{{\rm{Protection}}}}}}\,{{{{{\rm{ratio}}}}}}\,( \% )=1-{{{{{\rm{silica}}}}}}\,{{{{{\rm{coverage}}}}}}\,{{{{{\rm{ratio}}}}}}\,( \% )$$

Figure 1c−e and Supplementary Fig. 5 present the TEM images of core–shell structured NaErF_4_/SiO_2_@NaYF_4_ Janus nanoparticles with different protection ratios of NaYF_4_ shell (25%, 75%, and 100% (NaErF_4_@NaYF_4_)), which show that the as-prepared Janus nanoparticle consists of the following parts: the NaErF_4_ UCNP core, the SiO_2_ shell covering one side of the core, and the NaYF_4_ shell on the other side.

### Spectroscopic study of core-shell structured NaErF_4_/SiO_2_@NaYF_4_ Janus nanoparticles

Figure 1f compares the upconversion luminescence intensities of core-shell structured NaErF_4_/SiO_2_@NaYF_4_ nanoparticles under 980 nm laser excitation (3 W cm^−2^) with different protection ratios of NaYF_4_ shell, i.e., 25% protection (NaErF_4_/SiO_2_@25%NaYF_4_), 75% protection (NaErF_4_/SiO_2_@75%NaYF_4_), and 100% protection (NaErF_4_@NaYF_4_). The bare NaErF_4_ core nanoparticle (0% protection) serves as the control group for comparison. Surprisingly, with the protection ratio of NaYF_4_ shell increased from 0% to 25%, and to 75%, the upconversion luminescence intensity remains almost unchanged in the spectral range from 500 nm to 700 nm (Fig. 1g). Only when a full NaYF_4_ shell (100% protection) is deposited on the NaErF_4_ core, the luminescence intensity can be recovered and dramatically improved, by about 2500 times (Supplementary Figure 6). Previous work showed that the inert shell could act as a shield to protect the luminescent core from surface quenchers^28^. In that case, evident enhancement in luminescence intensity should be anticipated when more surface defects are passivated with the protection of NaYF_4_ shell. However, our results clearly suggest that the luminescence intensity of UCNP core does not synchronously improve with continuous passivation of exposed surface defects. Instead, an “off-on” effect exists at the core-shell interface, meaning that partial coverage of inert shell to any degree contributes little to the luminescence enhancement, and a full coverage with the inert shell is critically necessary.

In lanthanide-doped nanocrystals, the luminescence emission intensity would decrease when the dopant ion concentration exceeds a threshold, typically referred to as “concentration quenching”. It has been well established that an inert shell could reduce the coupling between surface and concentration quenching effects via suppressing energy migration to the surface quenchers^29,33^. Figure 1h and i present the upconversion luminescence decay curves of green emission (542 nm) and red emission (658 nm) for NaErF_4_/SiO_2_@NaYF_4_ Janus nanoparticles with different protection ratios of NaYF_4_ shell. It can be observed that the upconversion luminescence lifetime for both green and red emissions generally increase with the increased protection ratios of NaYF_4_ shell. The enhancement in lifetime does indicate longer time of excitation states remaining in the system when more surface defects are gradually recovered by the coating of NaYF_4_ shell. However, as mentioned in Fig. 1f and g, the upconversion luminescence intensity exhibits no evident enhancement with the increase of NaYF_4_ shell coverage only until the NaErF_4_ core surface is fully protected. Generally, for the NaErF_4_ core nanoparticle, some internal defects (e.g., vacancy and interstitial defects) may exist and quench the luminescence (Fig. 1j). Besides, when the size of the particle decreases, the total surface area per volume will increase because of the higher surface-to-volume ratio, which promotes surface-related deactivations due to more exposure to the surface defects (e.g., crystal disorder and non-crystallization) as well as capping ligands and solvent molecules with strong vibrations^34,35^. In this regard, these surface-related quenching processes can affect the luminescence of as-synthesized NaErF_4_/SiO_2_@NaYF_4_ Janus nanoparticles via following routes^36^ (Fig. 1j):

i. Direct deactivation. Photoexcited Er ions located on (or close to) the particle surface are non-radiatively deactivated by the neighboring quenchers.
ii. Energy migration induced quenching. This refers to the quenching process, in which the excitation energy of internal photoexcited Er ions randomly migrates to other Er ions around the surface or directly to the surface quenching site.

Notably, as these surface-related processes can be considered as a form of Förster resonance energy transfer (FRET)^37^, the FRET efficiency via dipole-dipole coupling significantly depends on the separation distance between energy donor and energy acceptor with an inverse 6^th^ power law:

2 $$E=\frac{1}{1+{(\frac{r}{R})}^{6}}$$

where *E* is the efficiency of energy transfer, *R* is the Förster distance and *r* is the distance between the pair of energy donor (i.e., photoexcited Er ion) and energy acceptor (quenching center). As a result, only when a full inert NaYF_4_ shell is coated on the NaErF_4_ core, the increased distance between luminescent core and surrounding quenching centers can greatly prevent the quenching of excited Er ions around the surface and also curb the migration of excitation energy to the surrounding quenchers.

In addition to shell coverage, we have further investigated the influence of thickness of incomplete shell on the luminescence intensity of NaErF_4_/SiO_2_@NaYF_4_ Janus nanoparticles. As shown in Supplementary Fig. 7a, with increasing the thickness of the incomplete NaYF_4_ shell (75% coverage) from 0 nm, to 5 nm and to 10 nm, the luminescence intensity still remains almost unchanged. This is a stark contrast to previous results that the luminescence intensity for core-shell UCNPs should generally exhibit a thickness-dependent behavior^29,30,38^. Also, other than NaErF_4_ core, we have additionally used the low-doped luminescent core, NaYF_4_:20%Yb, 2%Er, to study the influence of NaYF_4_ shell coverage on the luminescence intensity (Supplementary Fig. 7b). Similar to the case of NaErF_4_/SiO_2_@NaYF_4_, the luminescence intensity of NaYF_4_:20%Yb,2%Er/SiO_2_@NaYF_4_ still barely improves with increasing the coverage ratio of NaYF_4_ shell from 0 % to 75%. Only when a full NaYF_4_ shell is coated on the NaYF_4_:Yb, Er core, the upconversion luminescence intensity can then be recovered and drastically improved. In other words, the well-studied parameters such as shell thickness and dopant concentration in previous studies, have little influence on the luminescent intensity, provided that the luminescent core is not completely covered by the inert shell. Therefore, based on the results in Fig. 1 and Supplementary Fig. 7, it is rather clear that no matter how to tune the coverage and thickness of the outer shell as well as the dopant concentration, the luminescence does not change accordingly unless the surface of the core nanoparticle is fully encapsulated. The existence of such “off-on” effect could be due to the reason that as long as the luminescent core is not completely covered by the inert shell, the exposed surface can still act as weak spots where the majority of excitation energy is dissipated via the migration to the surface quenchers and results in little enhancement in emission intensity.

**Fig. 2:**
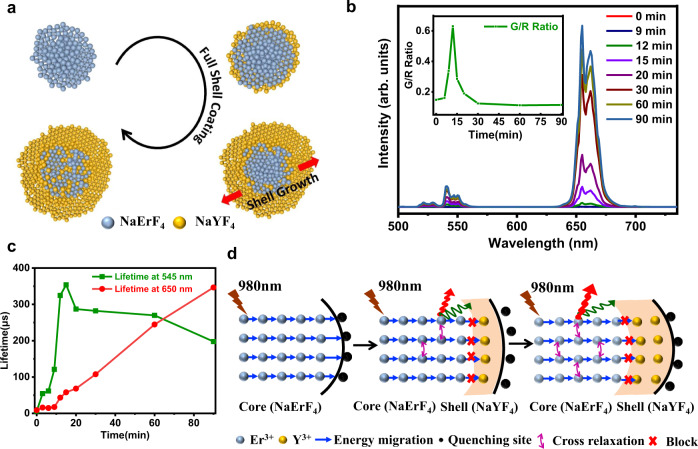
Time-course study on the growth of core-shell NaErF_4_@NaYF_4_ UCNPs. **a** Schematic of the growing process of full NaYF_4_shell (yellow) on the NaErF_4_ core (blue). During the full shell coating process, the NaYF_4_ shell would grow along both sides (red arrow) of the luminescent NaErF_4_ core. **b** The changes of upconversion emission spectra under 980 nm laser light (3 W cm^−2^) over time. Inset is the variations of green to red ratio over time. **c** The lifetime of red and green emissions over time. **d** The mechanism of the surface protection of NaErF_4_ core by NaYF_4_ shell with different thickness. Upon excitation by 980 nm light, the excitation energy is rapidly transferred to the surface quenchers for bare NaErF_4_ core. When a thin NaYF_4_ shell (shaded region) is coated, the surface defects are partly restored that preferentially promotes the green emission. As the thickness of NaYF_4_ shell goes up, the energy loss from Er^3+^ ions to surface defects is further blocked and the possibility of cross relaxation between Er^3+^ ions is greatly raised, which dramatically enhances the red emission.

To further understand the evolution of luminescent profiles with the coating of NaYF_4_ shell on the NaErF_4_ core, we have performed a time-course study on the growth of core-shell structured NaErF_4_@NaYF_4_ UCNPs (Fig. 2a). Our strategy was to start with the bare NaErF_4_ core in the solution, and then mix into the shell precursor to initiate the epitaxial growth of NaYF_4_ shell. Once the reaction is started by heating the mixture to required temperature, we sampled at different time intervals throughout the synthesis process. In Fig. 2b, upconversion spectra of NaErF_4_ core and core-shell NaErF_4_@NaYF_4_ UCNPs upon excitation by 980 nm laser light (3 W cm^−2^) are presented for samples obtained at different time points. Over the time course, there is a general increase in the emission intensity of upconversion spectra. In order to make a more direct comparison, the ratio of green to red band (G/R) at each of the time course points are plotted in the inset in Fig. 2b. In terms of the G/R values, there are no significant differences among samples taken at 0 min, and 9 min, but a sharp increase was observed in the subsequent time points. Specifically, the G/R value maximizes (>0.6) at 12 min, and then abruptly drops until 30 min point and the subsequent G/R values remain quite stable over the rest of the time course. Figure 2c presents the lifetime decays of green and red upconversion luminescence of NaErF_4_ core and core-shell NaErF_4_@NaYF_4_ UCNPs at different time points. The average decay time of the green upconversion luminescence of the NaErF_4_ core is calculated to be only 6.1μs, which suggests the excitation energy is non-radiatively lost due to the rapid energy migration to the surface quenchers. On the contrary, the lifetimes of green emission for core-shell NaErF_4_@NaYF_4_ UCNPs are significantly increased. This indicates that the nonradioactive process previously occurring on the bare NaErF_4_ core is now greatly suppressed by the protection of the inert NaYF_4_ shell. Similar to green emission decays, the lifetime of red upconversion luminescence of core-shell NaErF_4_@NaYF_4_ UCNPs is also much longer than that of core NaErF_4_. These results reconfirm that the surface quenching dominates the energy loss process. Another interesting observation is that the lifetime for the red emission monochromatically increases over time, while the lifetime for the green emission increases initially but starts to decrease since the time point of 12 min. The decrease of lifetime for the green emission is an indication of the enhancement of the cross-relaxation process between Er^3+^ ions over time, i.e., ^4^F_7/2_ + ^4^I_11/2_ → ^4^F_9/2_ + ^4^F_9/2_ (Supplementary Fig. 8), since this leads to depopulation of green emitting level ^4^F_7/2_ and the population of red emitting states ^4^F_9/2_.

It should be noted that the Er^3+^ ions in the NaErF_4_@NaYF_4_ core-shell UCNPs have dual functions, as both sensitizers and activators. On one hand, the Er^3+^ ions can directly harvest the incident 980 nm laser light as sensitizers. On the other hand, the excited Er^3+^ ions can radiatively transit to the ground states through releasing green and red lights. As shown in Fig. 2d, for the bare NaErF_4_ core, its outer surface is exposed to a large amount of quenching centers. Upon illumination by 980 nm laser light, the energy transfer (ET) process from Er^3+^ to surface quenchers can be readily established, which significantly lowers the emission intensity due to the substantial energy loss on the surface. However, once the full inert NaYF_4_ shell starts to grow on the NaErF_4_ core, the Er^3+^ ions are protected from the surface quenchers by the NaYF_4_ shell. When the thickness of NaYF_4_ shell is low (~1 nm)^39^, the surface defects are partly respired, which preferentially promotes the green emission and causes the increase of G/R value. As the thickness of NaYF_4_ shell goes up, the energy loss by the ET process from Er^3+^ ions to the surface defects is further prohibited, resulting in the prominent preservation of excitation energies. Under such circumstance, the absorbed excitation power is high enough to maintain the active energy transfer between neighboring Er^3+^ ions and may lead to adequate populations in the ^4^I_11/2_ and ^4^F_7/2_ states. Therefore, the possibility of cross relaxation (CR) process between ^4^I_11/2_ and ^4^F_7/2_ states is greatly raised and the number of ions in the ^4^F_9/2_ states is accordingly increased, which dramatically enhances the red emission (Supplementary Fig. 8).

### Tuning surface energy dynamic via cation exchange

**Fig. 3:**
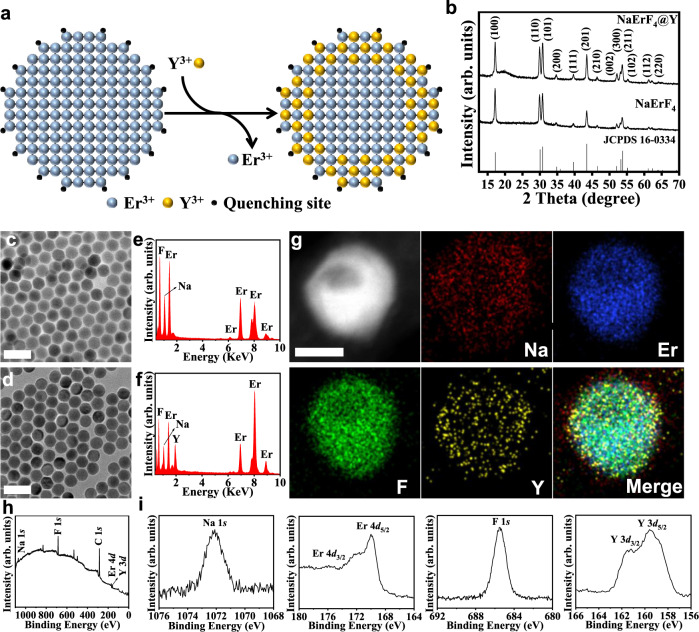
Structural characterization of NaErF_4_ nanoparticles before and after ion exchange. **a** Schematic of the NaErF4 core after ion exchange. The Y^3+^ ions (yellow) would replace some Er^3+^ ions (blue) on the surface and block the energy transfer to the quenching site. **b** X-ray diffraction (XRD) pattern of the NaErF_4_ nanoparticles, obtained before and after exchange with Y^3+^ ions (abbreviated as NaErF_4_@Y). **c**, **d** TEM image of the NaErF_4_ nanoparticles and NaErF_4_@Y nanoparticles, respectively. Scale bar: 50 nm. **e**, **f** The EDS line-scan profile of NaErF_4_ and NaErF4@Y nanoparticles, respectively. **g** HAADF-STEM (high angle annular dark field scanning TEM) of single NaErF_4_@Y nanoparticle and its corresponding elemental mapping images, Scale bar: 10 nm. **h**, **i** Full survey XPS spectrum and detailed spectra (Na 1 *s*, Er 4*d*, F 1 *s*, and Y 3*d*) of NaErF_4_@Y nanoparticles.

In light of the dominant role of surface-involved energy transfer in controlling luminescent profiles of UCNPs, we moved forward to optimize upconversion intensity by tailoring interfacial energy dynamics via a facile post-treatment. Specifically, a high-temperature ion exchange method is used to enhance the luminescence emission of UCNPs (Fig. 3a). In brief, pre-synthesized NaErF_4_ nanoparticles were mixed with YCl_3_ solution at elevated temperature (300°C) in order to obtain a new composite via cation exchange process, NaErF_4_@Y. Although surface cations on the nanoparticles are replaced by Y^3+^ ions, the hexagonal structure of NaErF_4_ phase can still be maintained (Fig. 3b). Besides, the TEM images and the size distribution analysis also reveal that the morphology and size of the NaErF_4_ nanoparticles do not significantly change during the ion exchange (Fig. 3c, d; Supplementary Fig. 9). The energy-dispersive X-ray spectroscopy (EDS) spectra (Fig. 3e and f) also confirm the presence of Y element for cation-exchanged NaErF_4_@Y nanoparticles. Moreover, elemental mapping images of a single NaErF_4_@Y nanoparticle show that Na, Er, and F elements are uniformly distributed on the nanoparticles while Y elements are mainly located on the outmost layer, indicating that ion exchange mainly occurs on the surface (see Fig. 3g). In addition, we have also used X-ray photoelectron spectroscopy (XPS) to analyze the chemical composition of NaErF_4_@Y nanoparticles before and after cation exchange. As shown in Fig. 3h,i, the signals from Y elements can be clearly identified from NaErF_4_@Y. However, no Y signals are observed from the XPS spectra of NaErF_4_ nanoparticles (Supplementary Fig. 10). It is worth mentioning that all the obtained nanoparticles have similar lattice constants compared with NaErF_4_ nanoparticles (Supplementary Table 1).

**Fig. 4:**
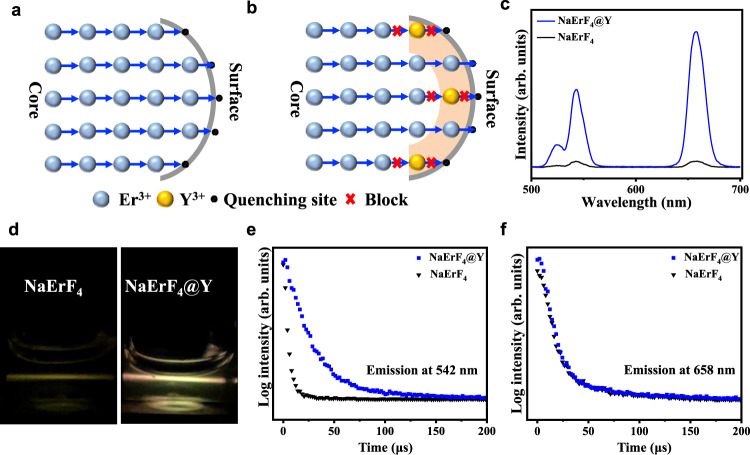
Spectroscopic study of NaErF_4_ before and after ion exchange. **a** Schematic diagram of luminescence quenching caused by energy transfer to the surface. The excitation energy would migrate among Er ions (blue) and travel to the quenching site (dark) on the surface. **b** Proposed mechanism of luminescence enhancement after ion exchange. The Y^3+^ ions replaced some Er^3+^ ions on the surface and created an effective shell region (shaded area) such that the energy migration to the surface quenching site was blocked. **c**, **d** Upconversion emission spectra and corresponding luminescence photographs of the NaErF_4_ and NaErF_4_@Y nanoparticles upon 980 nm laser excitation (3 W cm^−2^). **e**, **f** Lifetime decay curve of Er^3+^ emission at 542 nm and 658 nm from the NaErF_4_ and NaErF_4_@Y nanoparticles upon 980 nm laser excitation (3 W cm^−2^).

For a bare NaErF_4_ nanoparticle, as its surface is exposed to the surrounding quenchers, the upconversion luminescence intensity is severely reduced, due to the substantial transfer of the excitation energy to the surrounding quenchers (Fig. 4a). However, we reason that cations on the surface of NaErF_4_ nanoparticles are replaced by inert Y^3+^ ions through ion exchange so that the pathway of energy migration to surface quenchers can be blocked, which is expected to enhance upconversion luminescence (Fig. 4b). Figure 4c compares the upconversion luminescence spectra of NaErF_4_ and NaErF_4_@Y nanoparticles under 980 nm laser excitation (3 W cm^−2^). As anticipated, the luminescence intensity of NaErF_4_@Y nanoparticles is obviously stronger than that of NaErF_4_ nanoparticles, with an enhancement of about 17.2 times. Figure 4d presents the luminescence photos of NaErF_4_ and NaErF_4_@Y nanoparticles upon illumination by 980 nm light, which also confirms that the luminescence of NaErF_4_@Y is significantly brighter than that of NaErF_4_. Similar behavior can also be observed for the luminescence decay of Er^3+^ emission at 542 nm under 980 nm excitation (Fig. 4e). As expected, the lifetime value is increased from 5.6 µs to 28.6 µs. Notably, the lifetime of Er^3+^ emission at 658 nm remains almost unchanged (Fig. 4f), indicating that the exchange of NaErF_4_ nanoparticles with Y^3+^ ions has a greater influence on the green emitting state.

To further confirm that the cation substitution with Y^3+^ ions is the main cause of luminescence enhancement, pre-synthesized NaErF_4_ nanoparticles were preheated for 1 h at the same temperature to exclude the temperature influence. As shown in Supplementary Figs. 11 and 12, the reheated NaErF_4_ nanoparticles (NaErF_4_@Reheating) can maintain the original shape, size, composition and phase structure. It can also be observed the upconversion emission of NaErF_4_ nanoparticles before and after reheating basically remains constant (Supplementary Fig. 13), indicating that only reheating treatment can not enhance the luminescence intensity NaErF_4_ nanoparticles.

In addition, Yb^3+^ ions are also used for ion exchange with NaErF_4_ nanoparticles to obtain a new composition of nanoparticles (NaErF_4_@Yb) to verify whether it has the same luminescence enchantment effect. The characterization in Supplementary Fig. 14 and Supplementary Fig. 15 confirm that Yb^3+^ ions have been successfully incorporated into NaErF_4_ nanocrystals. However, unlike NaErF_4_@Y nanoparticles, the upconversion luminescence of NaErF_4_@Yb is barely enhanced compared with that of NaErF_4_ nanoparticles (Supplementary Fig. 16a). A possible explanation is that the energy levels of Yb^3+^ ions and Er^3+^ ions are greatly matched so that the energy migration of excitation energy to surface quenchers can not be blocked (Supplementary Fig. 16b).

## Discussion

For the lanthanide-doped upconversion nanoparticles, a common approach to alleviate surface-related quenching effects is to adopt the core-shell strategy that passivates the luminescent core with an optically inert shell^28^. Due to its great effectiveness and easy fabrication, core-shell engineering has drawn extensive attentions. For example, B. Richards et al. have systematically investigated the crystallographic structure at the interface of core-shell and core-multi-shell nanoparticles, demonstrating that the cation inter-diffusion occurs between core and shell materials during the synthesis^15,40^. Besides, they have recently proposed an energy-migration strategy that controls the spatial redistribution of harvested energy from one sensitizing zone to two different emitting zones, which allows for the color-tuning of small-sized upconversion nanoparticles (<20 nm) with a high quantum yield efficiency up to 3.5% (60 W cm^−2^)^41^. Moreover, other factors have also been verified to have pronounced influence on the luminescence intensity, such as shell thickness^30,38^, doping concentration^29,33^, refractive index of surrounding solvents^37,42^, synthetic routes^27,43^, etc.

Despite these great efforts, it should be noted that full-shell coating is generally utilized for constructing core-shell structures in these studies. In this context, the impact of this work lies in solving a long-neglected issue in core-shell nanoparticles-that is to probe the relationship between step-wise surface passivation and resulting luminescence change. Only by precisely controlling how shell grows on the luminescent core can we draw accurate conclusions about the passivation-luminescence relationships. In this work, using the surface-templating strategy, the growth of NaYF_4_ shell on the NaErF_4_ core can be finely controlled, as shown in Fig. 1a–e. Besides, this strategy can also be employed to fabricate other Janus nanoparticles with dual surface hydrophobicity/hydrophilicity for many potential applications, including nanomotor, multi-modal imaging, cancer therapy, etc. On the successful construction of NaErF_4_/SiO_2_@NaYF_4_ Janus nanoparticles, we have demonstrated an “off-on” effect at the core-shell interface: regardless of the coverage ratio and thickness of the inert shell as well as the doping concentration, the luminescence is not much changed unless the core surface is completely encapsulated. Therefore, this means that the key factor that influences most on the surface passivation of an individual UCNP nanoparticle is the minimum thickness of inert shell instead of the average value. In other words, if the core nanoparticle is not fully coated, it seems that the core surface is little passivated.

Furthermore, it is believed that such “off-on” effect is not only applicable to core-shell UCNPs, but to many other luminescent counterparts, which would also be rather helpful to better understand the interfacial energy dynamics of core-shell nanosystems.

In summary, the long neglected relationship between interfacial quenching dynamics and upconversion luminescence in core-shell UCNPs is elucidated through step-wise passivation of surface defects via a specially designed Janus architecture. Our results clearly indicate that full shell encapsulation on the luminescent core nanoparticle plays decisive roles in enhancing upconversion luminescence intensity. The time-course studies further show that both the upconversion luminescence intensity and color output vary with the thickness of full shell coverage. In light of this understanding, a non-core-shell strategy based cation exchange method is utilized to enhance upconversion luminescence of highly doped UCNPs, and demonstrate similar functionality as the core-shell approach. It is believed that our results offer important insights into the interfacial energy dynamics and may inspire new design of UCNP-based nanocomposites with optimized performance and broader application potentials.

## Methods

We synthesized upconversion nanoparticles using the method described in ref. ^31^. Additional experimental details are provided in the Supplementary Note.

### Synthesis of NaErF_4_/SiO_2_ Janus nanoparticles

Firstly, 3 mg mL^−1^ CTAB solution (10 mL) was mixed with 5 mg/mL NaErF_4_ in cyclohexane (1 mL), followed by 45 s of sonication using the Sonic Material Vibra-Cell Ultrasonic Processor (SciMed (Asia) Pte Ltd) until a milk-like emulsion formed. Then, the emulsion was transferred to a 25 mL one-neck round bottom flask and vigorously stirred at 1400 rpm and heated to 70 °C for 3 h until full removal of the cyclohexane. The solution would turn back to a clear state. After the solution was cooled down to room temperature, the NaErF_4_@CTAB solution (0.5 mg mL^−1^, mg mL^−1^) was transferred to another 25 mL one-neck round bottom flask. Then, 200 μL ammonium hydroxide solution was added into the flask and the solution was kept stirring under 1000 rpm. After 30 min, different amount of TEOS was added dropwise into the flask, during which the stirring speed was kept as 1400 rpm. After 3 h of stirring, the Janus nanoparticles were precipitated down by centrifugation and dispersed in 10 mL DI water and stored for further use. Notably, for the tuning of silica coverage, different amount of TEOS (15, 20, 25, 30, and 35 μL) were added into NaErF_4_@CTAB solution.

### Silica etching

10 mL NaErF_4_/SiO_2_ solution (0.5 mg mL^−1^) were mixed with specific amounts of BOE ranging from 5 to 12.5 μL to obtain NaErF_4_/SiO_2_ Janus nanoparticles with different SiO_2_ coverage ratio. The BOE solution was firstly diluted by 1 mL DI water to reduce the etching speed and achieve uniform etching morphology. The diluted BOE solution was added dropwise by the control a micro syringe pump at a speed of 1 mL h^−1^. Notably, the solution was kept for stirring under 1000 rpm. Then, the solution was centrifuged at 15000 rpm for 10 min and the obtained precipitates were dispersed in 10 mL ethanol.

### Preparation of core-shell structured NaErF_4_/SiO_2_@NaYF_4_ nanoparticles

Typically, based on the core-shell ratio, for every 10 mL ethanol containing etched NaErF_4_/SiO_2_ Janus nanoparticles, the aqueous solution of 0.03 mmol YCl_3_ was added into a 100 mL three-neck flask. After removing the water under 110 °C, the residuals were further dissolved in the mixture of 6 mL oleic acid and 15 mL 1-octadecene and the whole system was maintained at 156 °C for 10 min to completely form the RE-oleate complexes (RE: rare elements). Then, the mixture was cooled down to room temperature. The etched Janus NaErF_4_/SiO_2_ nanoparticles dispersed in 10 mL ethanol were added into the solution and the resulting mixture was heated to 120 °C to evaporate the ethanol. Upon cooling down to room temperature, followed was the addition of 150 μL methanol solution containing 0.12 mmol NH_4_F and 0.075 mmol NaOH. To remove the methanol, the temperature of the whole mixture was raised to 120 °C for 10 min. After that, to remove the oxygen and residual methanol, the system was degassed and filled by argon alternatively three times. Subsequently, the whole reaction was raised to 300 °C and kept for 1 h under the argon environment. The resulting solution was precipitated by adding the equal volume of acetone and centrifuged at 10000 rpm for 10 min. The precipitates were washed with acetone twice and finally dispersed in 10 mL cyclohexane and stored for further use.

### Synthesis of hexagonal NaErF_4_@Y UCNPs

NaErF_4_@Y UCNPs were obtained by ion exchange method. In a typical process, 1 mmol YCl_3_ aqueous solution was added to a 100 mL flask. The solution was heated at 120 °C to fully remove the water. Then, 6 mL of oleic acid and 15 mL of 1-octadecene were added to the flask and the mixture was heated to 155 °C until the solid powder is completely dissolved. The resulting solution was cooled down to room temperature. Subsequently, the pre-synthesized NaErF_4_ nanoparticles dispersed in cyclohexane (20 mL) were added. The resulting mixture was heated at 120 °C for 30 min to evaporate the cyclohexane. Then, the solution was heated to 300 °C under argon for 1 h and cooled down to room temperature. The products were precipitated by addition of acetone, collected by centrifugation, and washed with acetone several times. NaErF_4_@Yb UCNPs were synthesized by using an identical procedure, except for the use of YbCl_3_ in the synthesis.

### Synthesis of hexagonal NaErF_4_@Reheating UCNPs

In a typical process, the as-synthesized NaErF_4_ nanoparticles dispersed in cyclohexane (20 mL) were added. The resulting mixture was heated at 120 °C for 30 min to evaporate the cyclohexane. Then, the solution was heated to 300 °C under argon for 1 h and then cooled down to room temperature. The products were precipitated by addition of acetone, collected by centrifugation, and washed with acetone several times.

## Supplementary information

Supplementary Information

## Data Availability

The data that support the findings of this study are available from the corresponding author upon reasonable request. Source data are provided with this paper.

## Source data

Source data
