# Supplementary material for: Full shell coating or cation exchange enhances luminescence
Source: Nat Commun. 2021 Oct 26;12:6178. doi: 10.1038/s41467-021-26490-7 (PMC8548508; doi:10.1038/s41467-021-26490-7)
Supplement: Supplementary file 1 — Supplementary Information [file 41467_2021_26490_MOESM1_ESM.pdf]

SUPPLEMENTARY INFORMATION

Full Shell Coating or Cation Exchange Enhances Luminescence

Yi Zhang[1,#], Pengpeng Lei[1,#], Xiaohui Zhu[2,]*, Yong Zhang[1,]*

1. Department of Biomedical Engineering, Faculty of Engineering, National University of Singapore, Singapore City, Singapore 117583

2. School of Environmental and Chemical Engineering, Shanghai University, Shanghai, China, 200444

*To whom correspondence should be addressed, e-mail: X.Z. (xhzhu@shu.edu.cn), or Y.Z. (biezy@nus.edu.sg).

[#]These authors contributed equally.

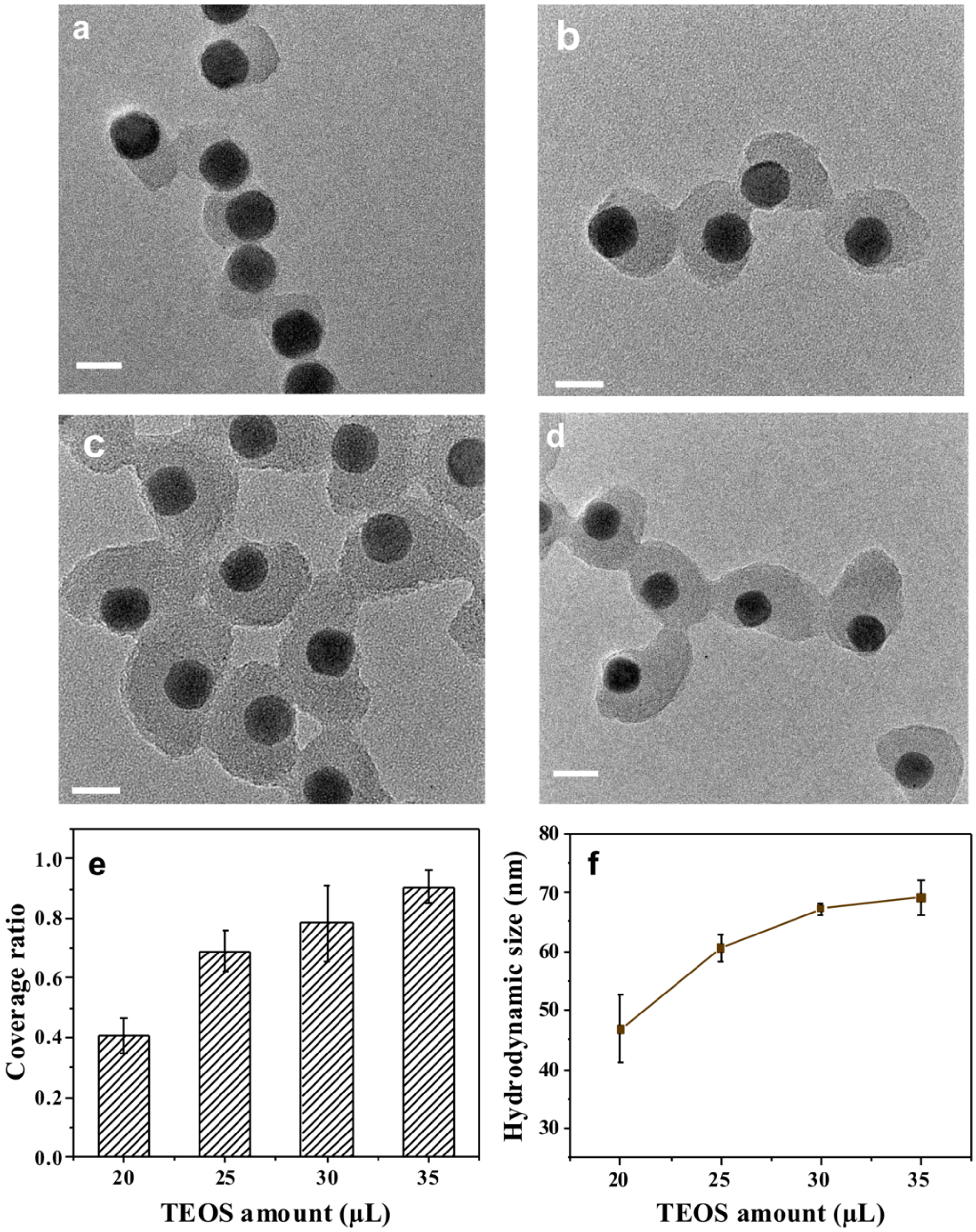

Supplementary Figure 1. Structural characterization of $NaErF_4/SiO_2$ Janus nanoparticles. TEM images showing the morphology control of the $NaErF_4/SiO_2$ Janus nanoparticles by adjusting the amount of TEOS: **(a)** 20 μL, **(b)** 25 μL, **(c)** 30 μL, and **(d)** 35 μL. Scale bar: 20 nm; **(e)** Calculated SiO_2 coverage ratio of $NaErF_4/SiO_2$ Janus nanoparticle with increasing TEOS amount; **(f)** Averaged hydrodynamic size of $NaErF_4/SiO_2$ Janus nanoparticles with increasing TEOS amount.

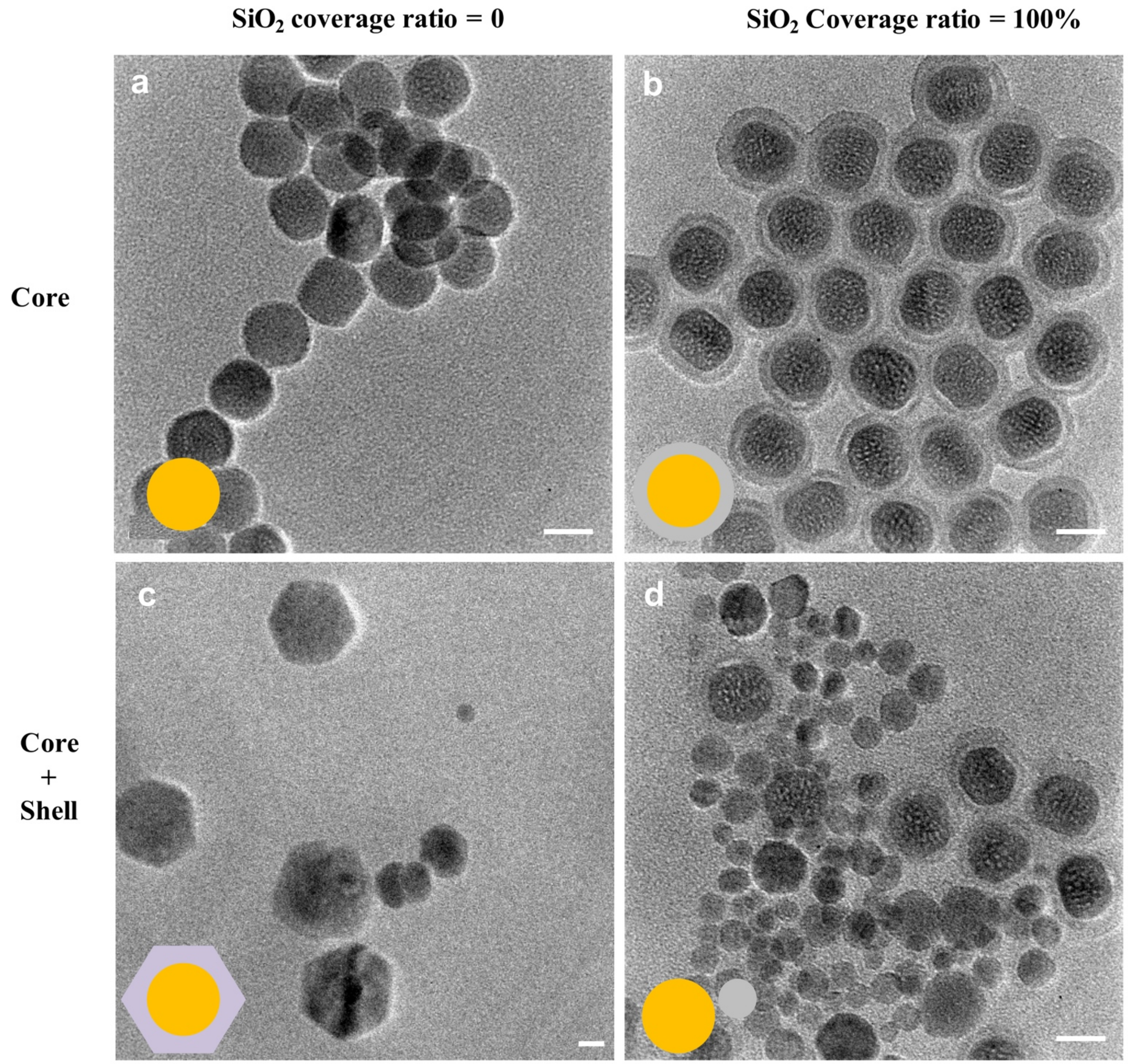

Supplementary Figure 2. Synthesis of $NaYF_4$ shell onto core $NaErF_4/SiO_2$ nanoparticles with 0% and 100% silica coverage. (a) and **(b)** show the TEM images of core $NaErF_4/SiO_2$ nanoparticles with SiO_2 coverage ratio of 0 and 100%. Scale bar: 20nm. **(c)** and **(d)** show The TEM images of $NaErF_4/SiO_2@NaYF_4$ nanoparticles with SiO_2 coverage ratio of 0 and 100%. Scale bar: (c) 50 nm, (d) 20 nm.

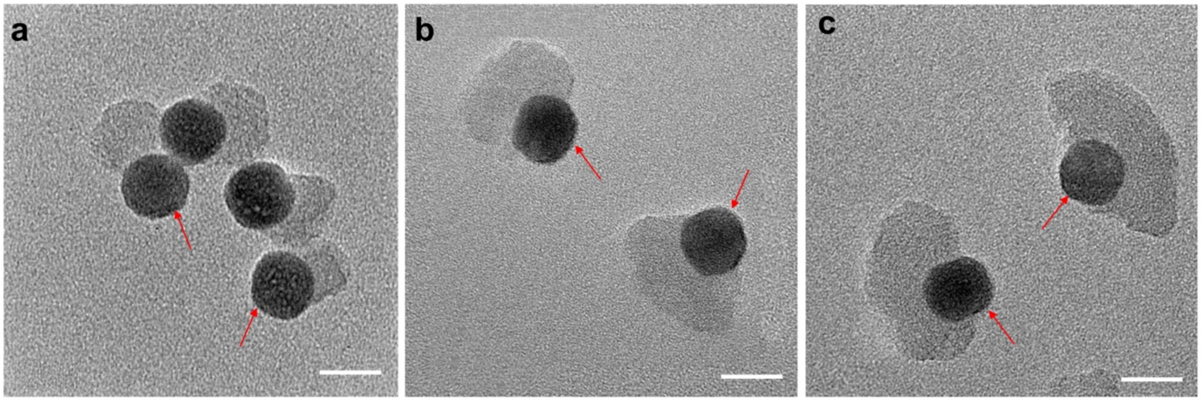

Supplementary Figure 3. Characterization of $NaErF_4/SiO_2$ Janus nanoparticles after BOE etching. The TEM images showing morphology and coverage ratio change, size and size uniformity of $NaErF_4/SiO_2$ Janus nanoparticles after BOE treatment: **(a)** 25% silica coverage, **(b)** 50% silica coverage, and **(c)** 75% silica coverage. The red arrow in each panel indicates the exposed surface of $NaErF_4$ nanoparticle after BOE treatment. Scale bar: 20 nm.

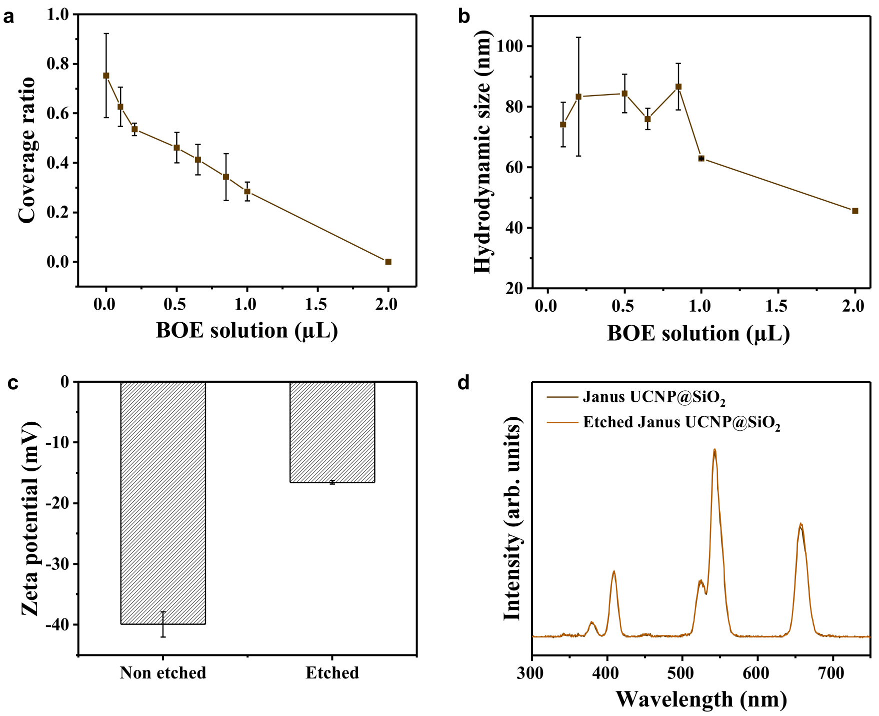

Supplementary Figure 4. Characterization of $NaErF_4/SiO_2$ Janus nanoparticles before and after etching. (a-b) The SiO_2 coverage ratio **(a)** and hydrodynamic size **(b)** of $NaErF_4/SiO_2$ nanoparticles etched by different amount of BOE solution. **(c)** The difference of zeta potential of $NaErF_4/SiO_2$ before and after BOE etching. **(d)** The emission spectra of $NaErF_4/SiO_2$ in water before and after etching upon the 980 nm laser irradiation.

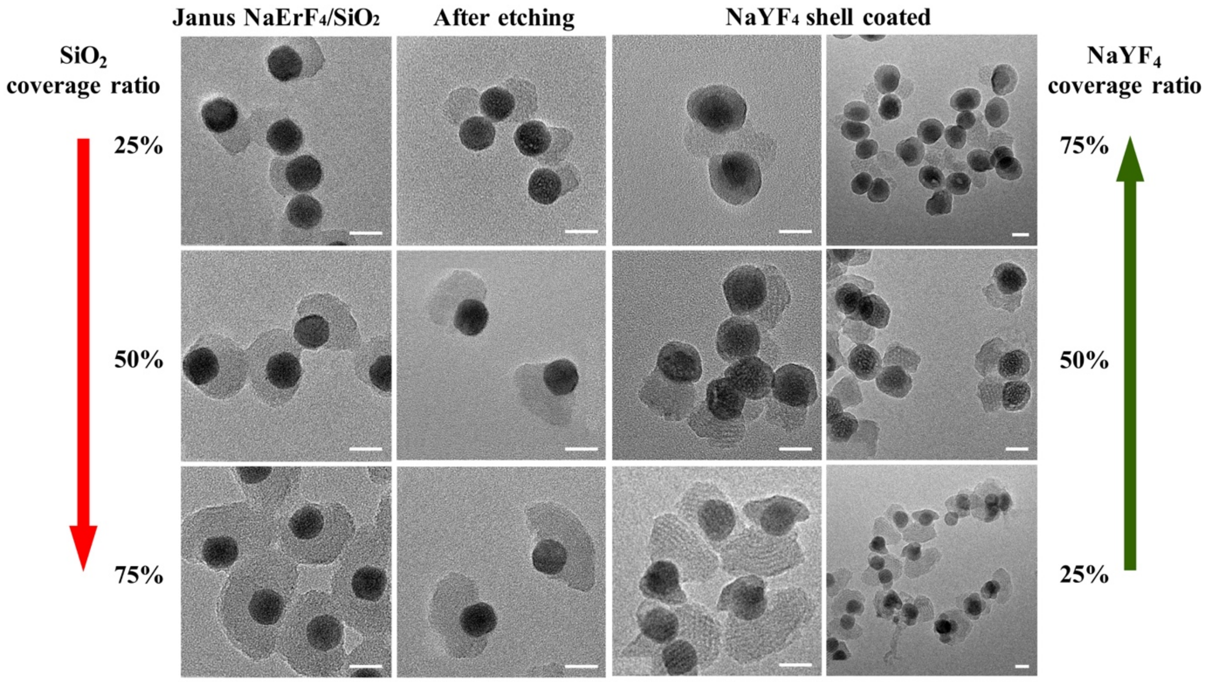

Supplementary Figure 5. Preparation of core-shell structured $NaErF_4/SiO_2@NaYF_4$ Janus nanoparticles. The TEM showing morphology, size and size uniformity of $NaErF_4@SiO_2$ nanoparticles, etched $NaErF_4@SiO_2$ and core-shell structured $NaErF_4/SiO_2@NaYF_4$ nanoparticles, with an increased silica coverage ratio, 25%, 50%, 75%. For core-shell structured $NaErF_4/SiO_2@NaYF_4$ Janus nanoparticles, 75%, 50% and 25% of the surface area were protected by inert $NaYF_4$ shell. Scale bar: 20 nm.

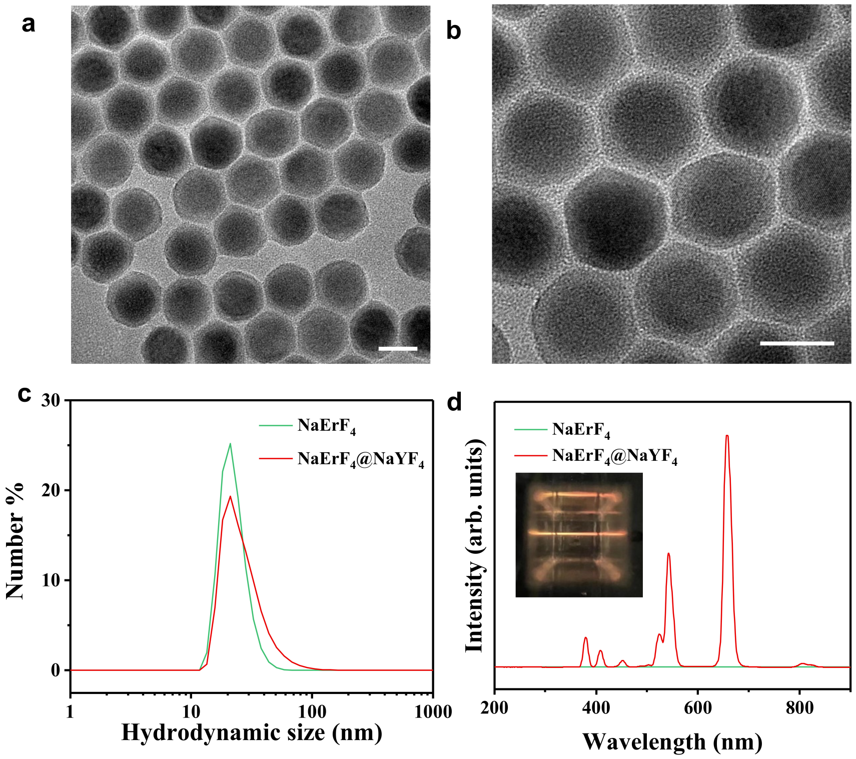

Supplementary Figure 6. Characterization of core-shell structured $NaErF_4$@$NaYF_4$ UCNPs. (a-b) The TEM showing size and size uniformity of $NaErF_4$@$NaYF_4$. Scale bar: 20 nm. **(c)** DLS showing hydrodynamic size and size distribution of as prepared $NaErF_4$@$NaYF_4$ and $NaErF_4$ in cyclohexane. **(d)** Emission luminescence spectrum of $NaErF_4$@$NaYF_4$ and $NaErF_4$ UCNPs upon excitation at 980 nm NIR laser (3W cm^{-2}). Inset is the camera picture of $NaErF_4$@$NaYF_4$ UCNPs.

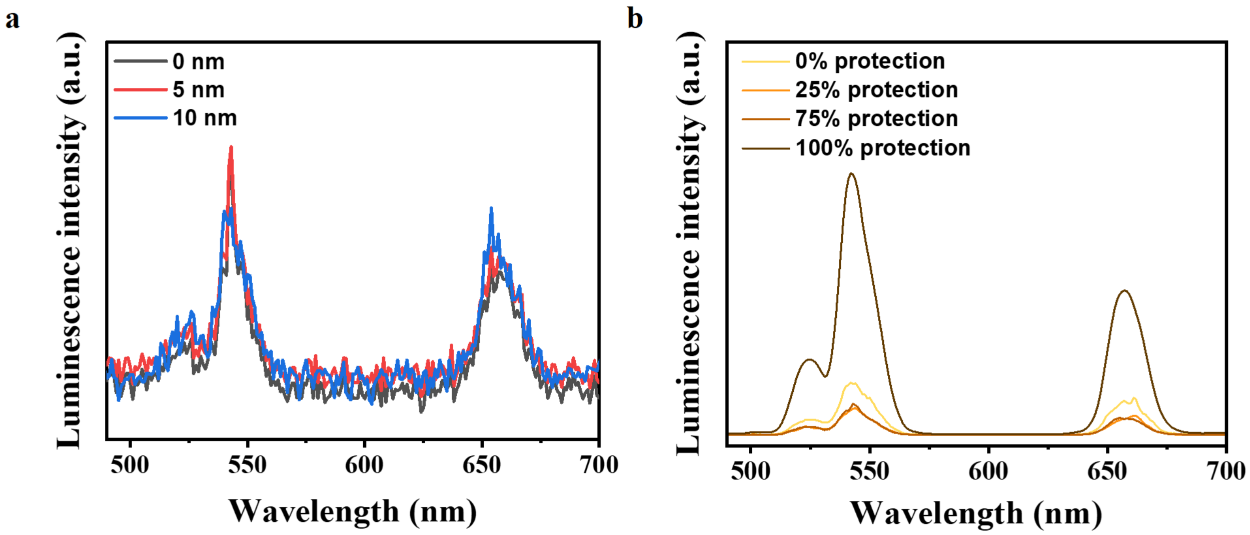

Supplementary Figure 7. "Off-on" effect for $NaErF_4/SiO_2$@75%$NaYF_4$ with different shell thickness and $NaYF_4$:Yb,Er/SiO_2@$NaYF_4$ UCNPs with different shell protection ratios. (a). The upconversion emission spectra of $NaErF_4/SiO_2$@75%$NaYF_4$ Janus nanoparticles with different thickness of NaYF4 shell (i.e., 0 nm, 5nm, and 10 nm) under 980 nm laser light excitation (3Wcm^{-2}). **(b)**. The upconversion emission spectra of $NaYF_4$:20%Yb,2%Er/SiO_2@$NaYF_4$ Janus nanoparticles with different shell protection ratios (i.e., 0%, 25%, 75% and 100%) under 980 nm laser light excitation excitation (3W cm^{-2}).

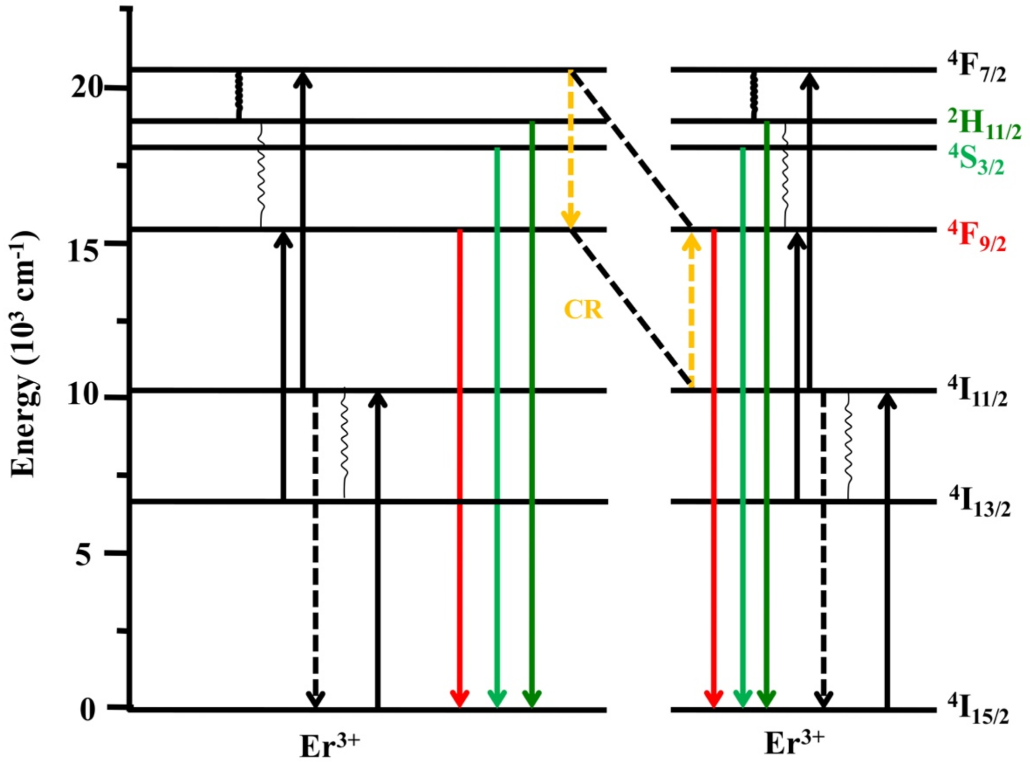

Supplementary Figure 8. Schematic illustration of the upconversion mechanism for $NaErF_4$ UCNPs under 980 nm excitation.

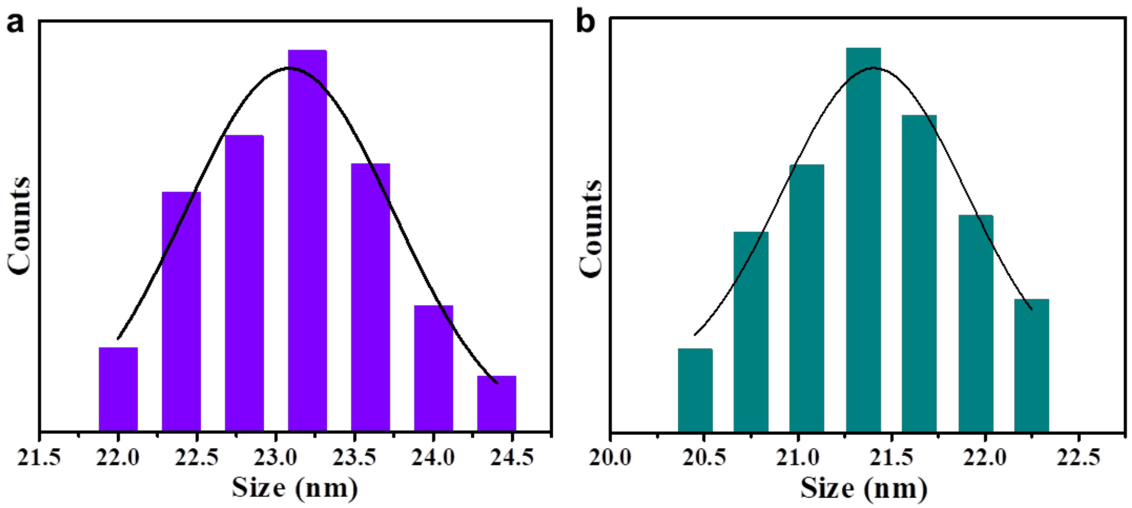

Supplementary Figure 9. Characterization of $NaErF_4$ nanoparticles before and after ion exchange. Size distributions of **(a)** $NaErF_4$ nanoparticles and **(b)** $NaErF_4$@Y nanoparticles.

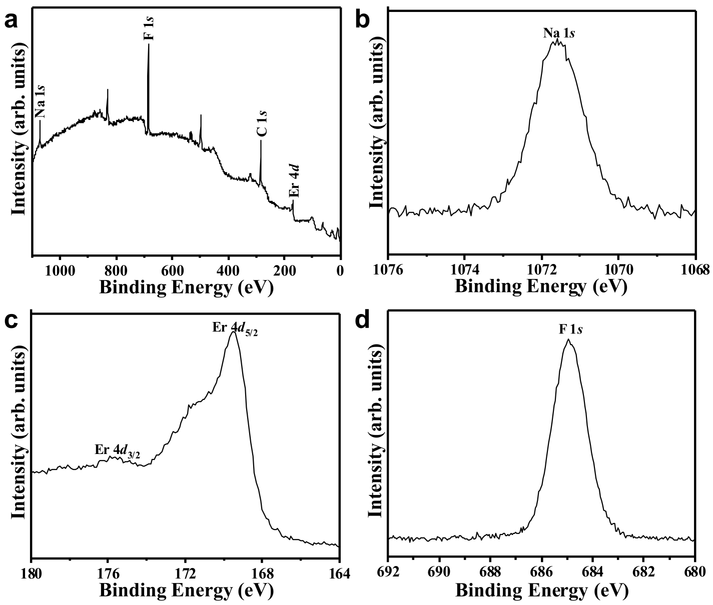

Supplementary Figure 10. Chemical analysis of $NaErF_4$ nanoparticles. XPS spectra of $NaErF_4$ nanoparticles. **(a)** Survey, **(b)** Na 1*s*, (c) Er 4*d*, and (d) F 1*s*.

Supplementary Table 1. Summary of the lattice constants of $NaErF_4$, $NaErF_4$@Y, $NaErF_4$@Reheating, and $NaErF_4$@Yb nanoparticles.

	a (Å)	b (Å)	c (Å)	α*β*γ
Standard XRD Card	5.959	5.959	3.514	90°*90°*120°
$NaErF_4$	5.968	5.968	3.512	90°*90°*120°
$NaErF_4$@Y	5.968	5.968	3.512	90°*90°*120°
$NaErF_4$@Reheating	5.970	5.970	3.510	90°*90°*120°
$NaErF_4$@Yb	5.968	5.968	3.510	90°*90°*120°

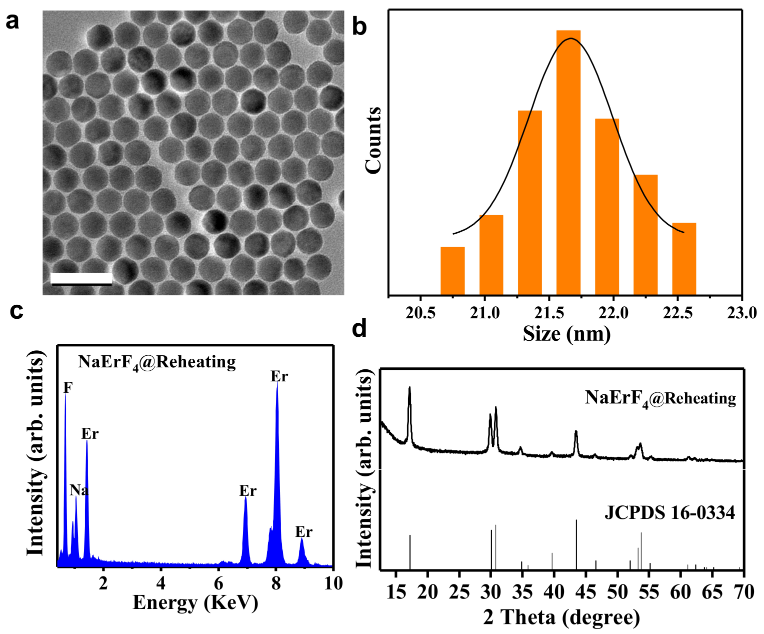

Supplementary Figure 11. Structural characterization of $NaErF_4$@Reheating nanoparticles. (a)TEM image, **(b)** corresponding size distribution, **(c)** EDS, and **(d)** XRD pattern of the $NaErF_4$@Reheating nanoparticles. Scale bar: 50 nm for panel a.

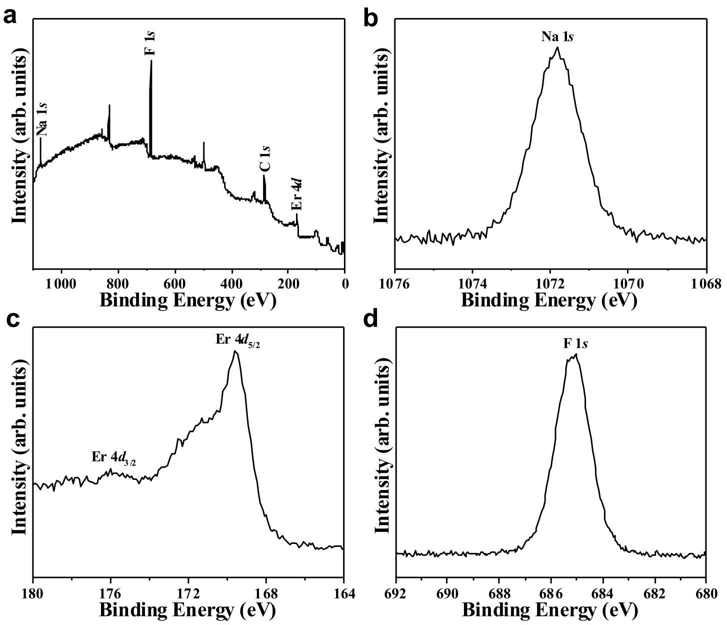

Supplementary Figure 12. Chemical characterization of $NaErF_4$@Reheating nanoparticles.

XPS spectra of $NaErF_4$@Reheating nanoparticles. **(a)** Survey, **(b)** Na 1*s*, **(c)** Er 4*d*, and **(d)** F 1*s*.

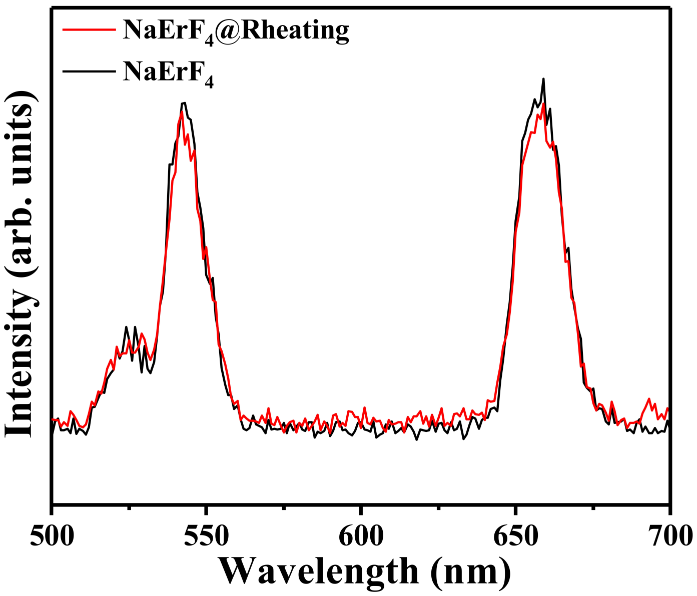

Supplementary Figure 13. Effect of reheating on the emissive profiles of $NaErF_4$ nanoparticles. Upconversion emission spectra of the $NaErF_4$ and $NaErF_4$@Reheating nanoparticles upon 980 nm laser excitation (3W cm^{-2}).

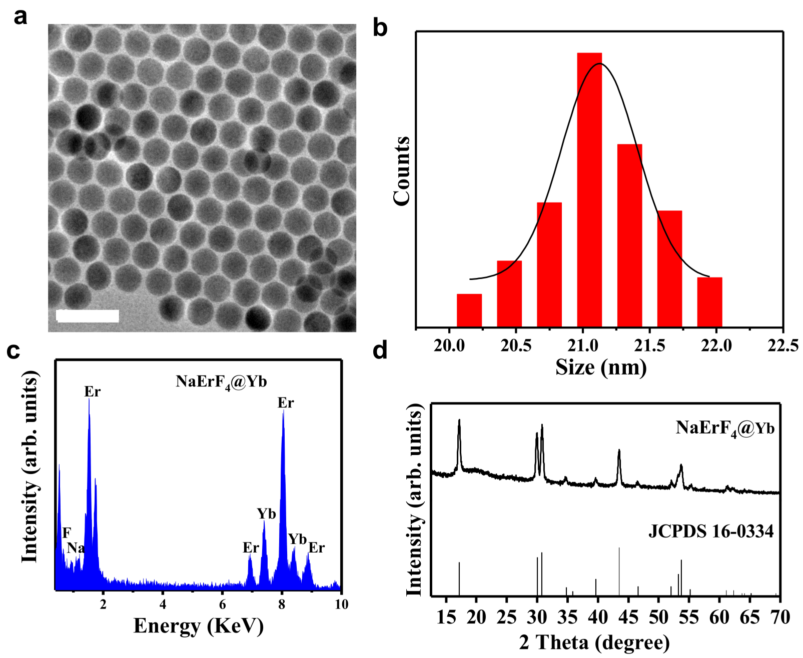

Supplementary Figure 14. Structural characterization of $NaErF_4$ nanoparticles after exchange with Yb^{3+} ions. (a)TEM image, **(b)** corresponding size distribution, **(c)** EDS, and **(d)** XRD pattern of the $NaErF_4$ nanoparticles after exchange with Yb^{3+} ions (abbreviated as $NaErF_4$@Yb). Scale bar: 50 nm for panel a.

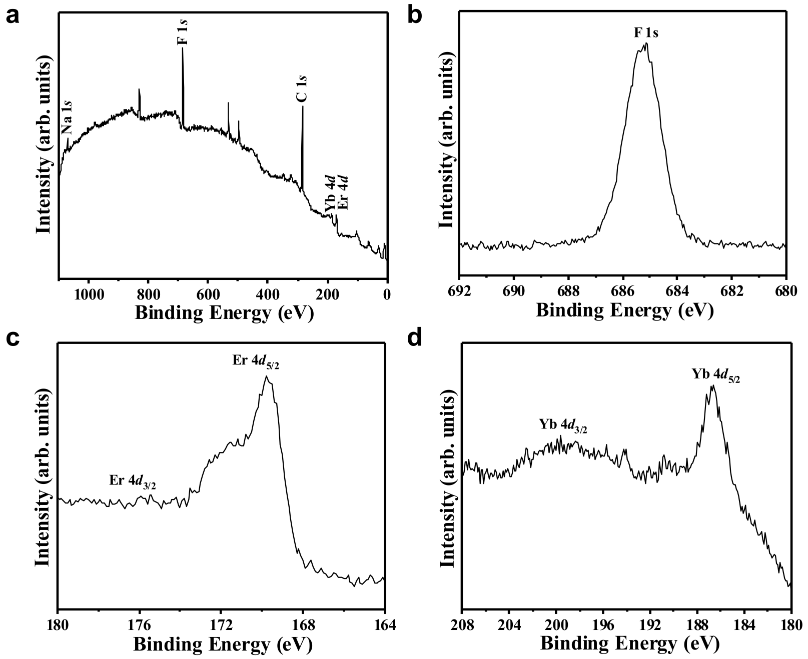

Supplementary Figure 15. Chemical characterization of $NaErF_4$ nanoparticles after exchange with Yb^{3+} ions. XPS spectra of $NaErF_4$@Yb nanoparticles. **(a)** Survey, **(b)** F 1*s*, **(c)** Er 4*d*, and **(d)** Yb 4*d*.

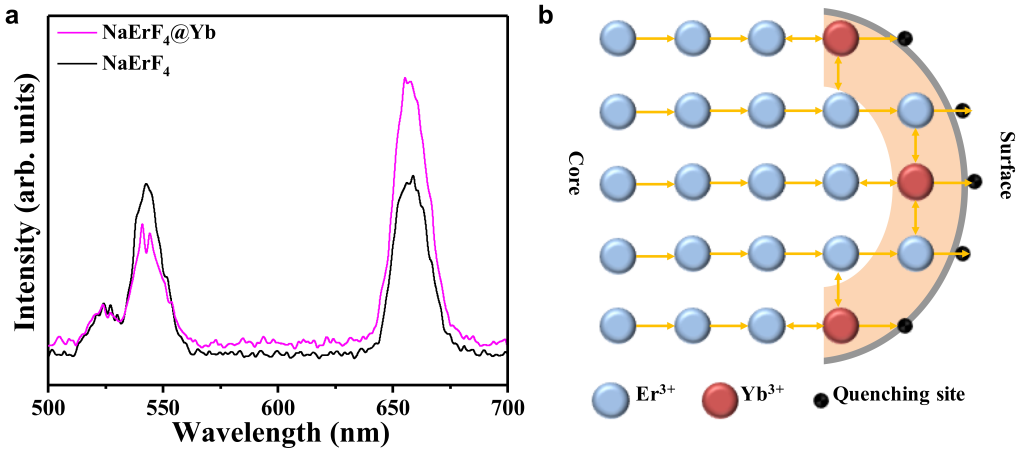

Supplementary Figure 16. Effect of Yb^{3+} exchange on the emissive properties of $NaErF_4$ nanoparticles. (a) Upconversion emission spectra of the $NaErF_4$ and $NaErF_4$@Yb nanoparticles upon 980 nm laser excitation (3W cm^{-2}). **(b)** Proposed mechanism of energy migration to surface defects of $NaErF_4$@Yb nanoparticles.

Supplementary Methods

Materials. Yttrium chloride (YCl_3•$6H_2O$, 99.9%), ytterbium chloride ($YbCl_3$•$6H_2O$, 99.9%), erbium chloride ($ErCl_3$•$6H_2O$, 99.9%), sodium hydroxide (NaOH, 98%), ammonium fluoride (NH_4F, 98%), 1-octadecene (90%), oleic acid (OA, 90%), cyclohexane, cetyltrimethylammonium bromide (CTAB), ammonium hydroxide solution ($NH_3 \cdot H_2O$, 33%), tetraethyl orthosilicate (TEOS, 98%), buffered oxide etchant (BOE) 10:1 solution, trimethoxy(octadecyl)silane (OTMS, technical grade) were purchased from Sigma-Aldrich and used as received without further purification.

Synthesis of $NaErF_4$ upconversion nanoparticles

In brief, 1 mmol $ErCl_3$ aqueous solution was added in a 100 mL three-neck flask. The mixture was stirred continuously at 300 rpm throughout the reaction and heated up to 110 ºC. After fully removing the water under 110 ºC, the residuals were dissolved in the mixture of 6 mL oleic acid and 15 mL 1-octadecene. Then, the whole system was maintained at 156 ºC for 10 min to completely form the RE-oleate complexes (RE: rare elements). Next, the mixture was cooled down to room temperature. 5 mL methanol solution containing 4 mmol NH_4F and 2.5 mmol NaOH was prepared and slowly added into the flask above and stirred for 30 min. To remove the methanol, the temperature of the whole reaction was raised to 120 ºC for 10 min. After that, the system was degassed and filled by argon alternatively three times to remove the oxygen and residual methanol. Subsequently, the whole mixture was raised to 300 ºC and kept for 1 h under the argon environment. The resulting solution was precipitated down by adding equal volume of acetone and centrifuged. The sediments were washed with acetone thrice to fully remove the residues and finally dispersed in 20 mL cyclohexane for further use.

Characterization

X-ray diffraction (XRD) measurement was performed with a D8 Focus diffractometer (Bruker) equipped with monochromatized Cu Kα radiation (λ = 1.5418 Å). Transmission electron microscopy (TEM) images and energy-dispersive X-ray spectroscopy (EDS) were obtained using a JEOL 2010F transmission electron microscope (Jeol Ltd., Tokyo, Japan) operating at an 200 kV. Elemental mapping measurement was performed on a double-aberration-corrected FEI Titan Themis G2 60-300 scanning transmission electron microscope (STEM). X-ray photoelectron spectroscopy (XPS) measurement was carried out in a Kratos Axis Ultra^{DLD} X-ray Photoelectron

spectrometer. UCL spectra were recorded on a Hitachi F-500 fluorescence spectrophotometer equipped with an NIR continuous wave (CW) laser with emission at 980 nm. The decay curve measurements were performed on the high performence Vis-NIR spectrometer (EINST Kymera 328i) equipped with both continuous 980-nm NIR laser and pulsed flash lamps. All the measurements were performed at room temperature. DLS size distribution and surface zeta potential were achieved from Malvern Zetasizer Nano Series (Malvern Instruments Ltd, Worcestershire, UK).
